# Midterm results after arthroscopic femoral neck osteoplasty combined with labral debridement for cam type femoroacetabular impingement in active adults

**DOI:** 10.1186/s13018-023-03543-9

**Published:** 2023-01-27

**Authors:** Filippo Migliorini, Nicola Maffulli, Andreas Bell, Francesco Cuozzo, Frank Hildebrand, Christian David Weber

**Affiliations:** 1 Department of Orthopaedic, Trauma, and Reconstructive Surgery, RWTH University Hospital, Pauwelsstraße 30, 52074 Aachen, Germany; 2 Department of Orthopaedic and Trauma Surgery, Eifelklinik St. Brigida, 52152 Simmerath, Germany; 3 Department of Medicine, Surgery and Dentistry, University of Salerno, 84081 Baronissi, SA Italy; 4 Faculty of Medicine, School of Pharmacy and Bioengineering, Keele University, Stoke on Trent, ST4 7QB England; 5 Barts and the London School of Medicine and Dentistry, Centre for Sports and Exercise Medicine, Mile End Hospital, Queen Mary University of London, London, E1 4DG England

**Keywords:** Hip arthroscopy, Cam, FAI, Labral tear, Sports

## Abstract

**Introduction:**

Arthroscopic labral procedures are frequently undertaken in patients with femoroacetabular impingement (FAI). The role of arthroscopic femoral neck osteoplasty is well established, but less is known about labral procedures. This study evaluates the midterm efficacy and feasibility of arthroscopic osteoplasty with concomitant labral debridement for cam impingement in active adults.

**Methods:**

The present study was conducted according to the STROBE Statement. All 108 patients who underwent primary hip arthroscopy for cam type FAI combined with labral debridement were considered. Axial and anteroposterior plain radiographs of the pelvis were obtained preoperatively to identify the cam deformity and assess the presence of osteoarthritis, the lateral centre-edge angle, and the alpha angle. The ROM (flexion, extension, abduction, adduction, and intra/extra rotation) was evaluated. The following PROMs were administered: visual analogic scale (VAS), Tegner Activity Scale, non-arthritic hip score (NAHS), the international Hip Outcome Tool (iHOT-33) overall score, and related subscales: symptoms and functional limitations, sports and recreational activities, job-related concerns, and social, emotional, and lifestyle concerns.

**Results:**

At a mean of 2.0 ± 1.1 months, all 108 patients returned to their daily activities with no limitation. At a mean of 2.6 ± 1.4 months, all 108 patients were able to return to sport with no limitation. At 11.9 ± 2.1-month follow-up, no differences were reported in the range of motion compared to baseline: flexion (*P* = 0.3), extension (*P* = 0.09), abduction (*P* = 0.1), adduction (*P* = 0.3), internal rotation (*P* = 0.4), and external rotation (*P* = 0.6). At 72.8 ± 21.7-month follow-up, the mean VAS score was 1.7 ± 3.0, Tegner Activity Scale 3.5 ± 1.4, NAHS 92.1 ± 21.5, iHOT33 83.3 ± 27.2. At a mean of 21.7 ± 15.5 months following the index procedure, 14 of 108 patients had undergone total hip arthroplasty. No further revisions or complications were reported.

**Conclusion:**

Arthroscopic femoral neck osteoplasty with concomitant labral debridement for cam type of FAI in active adults yields the reliable results, with a rate of progression to total hip arthroplasty of 13% (14 of 108) at 3-year follow-up. All patients returned to their daily activities with no limitation 2 months postoperatively. Within 3 months, all patients returned to sport with no limitations. No complication related to the index procedure was reported, and no further arthroscopic procedures were necessary.

## Introduction

In femoroacetabular impingement (FAI), the bony morphology of the femur and acetabulum may produce lesions of the acetabular labrum and cartilage [1–3], accelerating the development of symptomatic hip osteoarthritis [4, 5]. The incidence of the FAI has been reported at 54.4 per 100,000 person years and has increased in the past few decades [6–9]. A definite consensus on the extent of arthroscopic correction necessary to ameliorate FAI has not been achieved [10, 11], and there is a wide spectrum of surgical options to manage labral tears [12]. Arthroscopic osteoplasty for FAI has been frequently combined with labral surgery [13–15]. Labral resection/debridement was originally performed to reduce pain and increase joint function, especially in patients with unstable and/or irreparable labral lesions, and reconstructive techniques have recently become more common [16, 17]. Currently, labral repair and reconstruction retain the biomechanical role of the labrum and should allow better chance for hip preservation [3, 18–22]. However, debridement of the unstable peripheral portion of the labrum is technically less demanding and may be associated with a lower rate of intraarticular adhesions [16, 23]. The comparison of early outcomes of arthroscopic labral repair versus debridement based on the UK Non-Arthroplasty Hip Registry dataset showed that both labral procedures were successful in significantly improving early functional outcome after hip arthroscopy, regardless of age or sex [24]. There were superior outcomes after labral repair in univariate analysis, but no significant superiority in a multivariate model. A systematic review of repair versus debridement for acetabular labral tears identified superior patients reported outcome for labral repair, with however no significant difference in the conversion rate to total hip arthroplasty (THA) at up to 10-year follow-up [25]. Though recently the management of FAI has become more evidence-based, consensus on the more appropriate management is still lacking. The present investigation evaluates the midterm efficacy and feasibility of arthroscopic femoral neck osteoplasty with concomitant labral debridement for cam type FAI in active adults. It was hypothesised that this procedure leads to satisfactory outcomes with low rate of midterm progression to THA.

## Methods

### Study set up

The present study was conducted following the principles of the Helsinki Declaration and was approved by the ethic committee of the RWTH Aachen University (project ID EK 305/13). The present study was conducted according to the Strengthening the Reporting of Observational Studies in Epidemiology: the STROBE Statement [26]. In May 2021, the databases of the RWTH University Hospital of Aachen, Germany, and of the University Hospital of Salerno, Italy, were accessed. All the patients who had undergone primary hip arthroscopy for cam type FAI combined with labral debridement were identified.

### Eligibility criteria

The inclusion criteria were: (1) active patients involved in recreational activities; (2) adults aged 25–55 years; (3) radiographic evidence of cam deformity; (4) alpha angle > 55°; (5) symptoms and pain characteristic of FAI. The exclusion criteria were: (1) lateral centred edge angle (LCEA) < 25° or > 39°; (2) advanced hip osteoarthritis (grade 3 according to the Tönnis classification); (3) previous hip surgery; (4) symptomatic chondral defects greater than 0.5 cm^2^ which require surgical management; (5) any hip deformity or malformation; (6) uncontrolled chronic disease; (7) patients involved in high contact sports or playing elite or professional sport; (8) patients unable to understand the nature of the treatment and the finalities of the study. Only patients who completed the last follow-up were included for analysis.

Recreational physical activity was defined as an activity of leisure, without competition or high contacts, accomplished for enjoyment, amusement, or pleasure. Values of LCEA > 39° indicate pincer type impingement, and values < 25° are suggestive of acetabular dysplasia [27]. Values of alpha angle ≥ 60° indicate cam morphology [28]. The Tönnis classification (Table 1) was used to evaluate the degree of osteoarthritis [29].

**Table 1:** Tönnis classification

Grade	Description
0	No sign of arthritis
1	Increased sclerosis, slight narrowing of the joint space, no or slight loss of head sphericity
2	Small cysts, moderate narrowing of the joint space, moderate loss of head sphericity
3	Large cysts, severe narrowing or obliteration of the joint space, severe deformity of the femoral head

### Surgical technique

All the surgeries were performed by experienced surgeons following the same surgical protocol. With the patient supine, the anterior superior iliac spine, the greater trochanter, and the patella were identified and used as anatomical landmarks. Following insertion of a cannula into the hip joint under fluoroscopic guidance and the injection of 40 mL of normosaline, the hip joint was distracted under fluoroscopic guidance. A guide wire was inserted through the cannula. An antero-lateral portal was produced using the guide wire, and the arthroscope was inserted. An anterior portal was produced, followed by the proximo-anterior and caudo-anterior portals. The intraarticular joint space was inspected and a selective debridement of the acetabular labral lesions was performed. Labral debridement was limited to the peripheral labral tear only, paying attention to not compromise the labral sealing mechanism. The labrum was of functional size after debridement, and its stability at its chondrolabral junction was tested. A capsulotomy with the ArthroCare was performed. The CAM lesion was initially debrided with the ArthroCare. Finally, the CAM was removed using an Acromionizer and further debrided with the ArthroCare. A radiographic control was performed to check whether complete resection had been accomplished. Following removal of the arthroscope, the skin was sutured in the usual fashion, and sterile wound dressings were applied. Patients were allowed partial weight bearing with crutches with 20 kg for 4 weeks, and routine thrombosis prophylaxis was administered using enoxaparin.

### Outcomes of interest

Axial and anteroposterior plain radiographs of the pelvis were obtained preoperatively to identify the cam deformity and assess the Tönnis grade, the lateral centred edge angle (LCEA), and the alpha angle [30, 31]. At admission, the following data were recorded: age, gender, duration of symptoms before surgery, side, and BMI (Kg/m^2^). Data concerning the duration of the procedure and length of hospitalisation were collected. Between 6 and 12 months postoperatively, patients were invited for face to face the clinical examination. Range of motion (ROM) (flexion, extension, abduction, adduction, and intra/extra rotation) was evaluated twice using a standard longarm goniometer (Baseline Plastic Goniometers, Fabrication Enterprises Inc., White Plains, NY 10602 (USA). The time to return to daily activities and sport were recorded. In May 2021, patients were invited to participate in the study. The following PROMs were administered: visual analogic scale (VAS), Tegner Activity Scale [32], non-arthritic hip score (NAHS), the international Hip Outcome Tool (iHOT-33) overall score, and related subscales: symptoms and functional limitations, sports and recreational activities, job-related concerns, and social, emotional, and lifestyle concerns [33].

### Statistical analysis

The software STATA/MP 14.1 (StataCorp, College Station, TX, USA) was used in all the analyses. For each endpoint, arithmetic mean and standard deviation were calculated. The overall significance was calculated using the *t* test, with values of *P* < 0.05 considered statistically significant.

## Results

### Patient recruitment

**Fig. 1:**
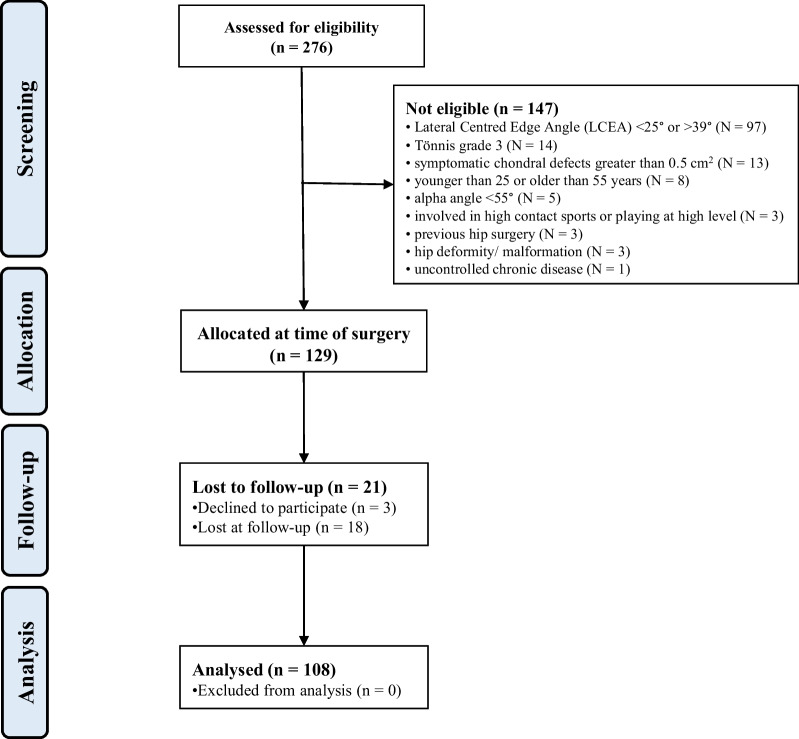
Patient recruitment

A total of 276 patients were initially enrolled. A total of 147 patients were excluded with reason: lateral centred edge angle (LCEA) < 25° or > 39° (*N* = 97), Tönnis grade 3 (*N* = 14), symptomatic chondral defects greater than 0.5 cm^2^ (*N* = 13), younger than 25 or older than 55 years (*N* = 8), alpha angle < 55° (*N* = 5), involved in high contact sports or playing at high level (*N* = 3), previous hip surgery (*N* = 3), hip deformity/malformation (*N* = 3), and uncontrolled chronic disease (*N* = 1). This left 129 eligible patients. Of them, three did not attend the follow-up between 6 and 12 months postoperatively and were therefore excluded from the analysis. A further 18 patients were lost to last follow-up. Finally, 108 patients were included in the present study (Fig. 1).

### Patient demographic at admission

43% (46 of 108 patients) were female, and in 59% (64 of 108 hips) the pathology was on the right side. The mean age of the patients at admission was 41.5 ± 14.8 years, and the mean BMI was 27.0 ± 4.8 kg/m^2^. The mean duration of symptoms before admission was 23.8 ± 22.6 months. On admission, 42% (45/108) of patients were classified as Tönnis 0, 52% (56/108) as Tönnis I, 6.5% (7/108) as Tönnis II. The mean alpha angle was 75.2° ± 12.0°, and the mean LCEA was 30.9° ± 5.1°. The mean length of hospitalisation was 3.7 ± 1.3 days. The mean duration of the surgical procedure was 79.1 ± 31.2 min. Patient demographic data are shown in Table 2.

**Table 2:** Demographic data of the patients at admission

Endpoint	Value
Last follow-up (months)	72.8 ± 21.7
Mean age	41.5 ± 14.8
Women	43% (46 of 108)
Right side	59% (64 of 108)
Symptom duration (months)	23.8 ± 22.6
Length of hospitalisation (days)	3.6 ± 1.1
Alpha angle (°)	75.2 ± 12.0
LCEA (°)	30.9 ± 5.1
BMI (kg/m^2^)	27.0 ± 4.8
Surgical duration (mins)	79.1 ± 31.5
*Tönnis grade*	
0	42% (45/108)
I	52% (56/108)
II	6.5% (7/108)
*ROM (°)*	
Flexion	112.3 + 13.2
Extension	3.0 ± 6.0
Abduction	35.8 ± 6.3
Adduction	25.5 ± 4.8
Internal rotation	23.8 ± 15.8
External rotation	34.6 ± 14.1

*LCEA* lateral centred edge angle, *ROM* range of motion)

### Outcomes of interest

Patients returned to their daily activities with no limitations at a mean of 2.0 ± 1.1 months. Patients were able to return to sport with no limitations at a mean of 2.6 ± 1.4 months. At 11.9 ± 2.1-month follow-up, no differences in range of motion were reported compared at baseline: flexion (*P* = 0.3), extension (*P* = 0.09), abduction (*P* = 0.1), adduction (*P* = 0.3), internal rotation (*P* = 0.4), and external rotation (*P* = 0.6). At 72.8 ± 21.7-month follow-up, the mean VAS score was 1.7 ± 3.0, Tegner Activity Scale 3.5 ± 1.4, NAHS 92.1 ± 21.5, iHOT33 83.3 ± 27.2.

### Complications

Fourteen of 108 patients underwent total hip arthroplasty of the operated hip at a mean of 21.7 ± 15.5 months. No complication related to the index procedure were reported, and no further arthroscopic procedures were necessary. These results are shown in detail in Table 3.

**Table 3:** Results at last follow-up

Endpoint	Value
Return to daily activities (months)	2.0 ± 1.1
Return to sport (months)	2.6 ± 1.4
VAS (0–10)	1.7 ± 3.0
Tegner Activity Scale	3.5 ± 1.4
NAHS	92.1 ± 21.5
*iHOT33*	
Symptoms and functional limitations	78.5 ± 25.7
Sports and recreational activities	85.0 ± 32.2
Job-related concerns	83.8 ± 28.7
Social, emotional, and lifestyle concerns	82.2 ± 31.8
Overall	83.3 ± 27.2
*ROM (°)*	
Flexion	127.0 ± 13.1
Extension	1.4 ± 4.7
Abduction	32.3 ± 11.4
Adduction	26.3 ± 11.5
Internal rotation	24.2 ± 15.6
External rotation	34.0 ± 11.4

*VAS* visual analogic scale, *NAHS* Non-arthritic hip score, *iHOT* International Hip Outcome Tool, *ROM* range of motion

## Discussion

The most important findings of this study are that arthroscopic femoral neck osteoplasty with concomitant labral debridement for cam type FAI in active adults is safe and feasible, with a reliable rate of success and a rate of progression to THA of 13% by 3-years of follow-up.

Lall et al. recently reported a consensus-based classification system for intra-operative management of labral teats during hip arthroscopy [34]. In this study, high-volume hip preservation surgeons suggested labral debridement for torn labra in viable (torn tissue that is likely to heal) and stable lesions for patients > 40 years of age (77%), and in stable intra-substance labral tears in patients (84%) without dysplasia (LCEA > 35°). Magnetic resonance arthrography may show new tissue formation in most patients who underwent hip labrum resection, and patients with labral refixation may present with irregular labrum shape (26.5%), rounded labrum shape (51.3%), or a triangular shape in less than 25% of cases [35].

Arthroscopy is a major advance in the management of symptomatic FAI. A recent systematic review evaluated at approximately 4 years of follow-up the clinical and functional outcomes of arthroscopy for FAI in 6510 procedures (6273 patients), [36]. Arthroscopy was associated with greater values of hip rotation and significant improvement in PROMs [36]. The rates of revision surgeries and progression to THA were 7.4% and 5.9%, respectively [36]. In patients with symptomatic FAI, arthroscopy yields better outcomes than conservative management [37]. Compared to open techniques, arthroscopy resulted in greater ROM and carries the advantage of being minimally invasive [38]. The reliable outcome of arthroscopy for FAI is also evident in adolescents [39]: 94% of adolescents had returned to sport at a mean of 28.0 months from the index procedure [39] All the PROMs of interest significantly increased, and 4.7% of adolescent patients necessitated a revision arthroscopy [39].

Return to sport, PROMs, complications, and subsequent progression to THA in patients undergoing arthroscopic labral repair for FAI have been recently investigated in a systematic review involving 240 procedures [18]. At approximately 38 months of follow-up, the rate of revision and progression to THA was 4.3% and 1.9% [18], and all patients had returned to sport within 2-yearsfollow-up [18]. However, in that systematic review pincer and mixed morphologies were also considered. Irrespective of labral procedures, arthroscopy for FAI demonstrated high rates of return to sport, remarkable improvement of PROMs, and a low rate of complications [40]. The rate of return to sport after arthroscopy for FAI has been also investigated in another systematic review including 41 studies (4063 procedures) [40]. 89% of patients returned to sport at a mean of 37 months. The Hip Outcome Score-Sport-Specific Subscale (HOS-SSS) improved considerably postoperatively [40]. In that study, younger age, reduced BMI, and good performance status preoperatively were associated with a quicker recovery and earlier return to sport [40]. Weber et al. evaluated return to sports (RTS) after hip arthroscopy for FAI in high-level athletes and reported an overall RTS rate of 90% [41]. The authors observed higher rates of labral debridement in contact sports athletes, but no difference in the RTS rate, duration of sport participation after return, or highest attempted level of sport resumed between patients who underwent labral repair versus debridement [41].

Labral preservation with repair procedures is considered the best available treatment for labral tears [42–45], but labral debridement yields very good outcomes in hip arthroscopy [46–50]. Isolated labral debridement was associated with improvement in PROMs and patients’ satisfaction and low rate of complications [50]. The outcomes of interest improved significantly from baseline to 2-year follow-up, and this improvement was confirmed at a minimum 5-year follow-up [50]. A systematic review of repair versus debridement for acetabular labral tears identified superior PROMs for labral repair, with no significant difference in the conversion rate to total hip arthroplasty (THA) at up to 10-year follow-up [25]. A recent investigation compared labral debridement versus repair in 73 hips [48]. At approximately 4-year follow-up, no difference was evidenced in ROM of the hip, activity of daily living subscale of the Hip Outcome Score and VAS [48]. Progression to THA was reported in two patients in the repair cohort, and in one patient in the debridement cohort [48]. One patient in the repair group underwent revision surgery for persistent joint instability [48]. Another investigation reported that, for select patients with stable labral tears, a selective debridement with labral preservation allows to maintain a functional labrum, with an improvement in PROMs comparable to those achieved in matched-pair labral repair group at a minimum 5-year follow-up [49].

This study certainly has limitations. The retrospective nature of the present investigation impacts negatively the risk of selection bias. The limited number of patients and the lack of randomisation may also impact the reliability of the conclusions. We acknowledge that some patients were lost to follow-up. The institution in which the operations were conducted is a tertiary referral centre for these pathologies, attracting patients from the whole country. Therefore, many patients were not able to undertake a long journey for research purposes. Moreover, the last follow-up was conducted during the Covid-19 pandemic, which considerably limited the ability of patients to travel to participate to the study. All the patients underwent conservative management prior to the arthroscopic procedure; however, given the heterogeneous nature and/or the lack of documentation on the previous conservative management, it was not possible to analyse the effects of such conservative modalities on the eventual outcome of these procedures. The lack of a control group and the unblinded design of the study represent additional limitations. However, all the patients were evaluated pre- and postoperatively in a strict fashion and were followed up using validated questionnaires. During the study, the joint capsule management did depend on capsular integrity, so a (partial) repair with non-absorbable suture applied by a suture passer were performed in cases with large (T-shaped) capsulotomy or observed instability. Patients with stable hips or controlled interportal capsulotomy or limited capsulotomy by the zona orbicularis did not undergo capsular repair. We acknowledge that criteria for patient selection including middle-aged patients with obesity on the one hand, and also the time span of observation between the onset of symptoms (2 years prior to surgery) and the extended follow-up may both have contributed to a significant rate of captured treatment conversions to THA, but this reflects clinical life in real practice and strengthens the generalisability of the results reported in the present investigation.

## Conclusion

Arthroscopic femoral neck osteoplasty with concomitant labral debridement for cam type of FAI in active adults yields reliable results and is safe, with a substantial rate of progression to THA of 13% at 3-year follow-up. All patients returned to their daily activities with no limitation 2 months postoperatively. Within 3 months, all patients had returned to sport with no limitation. No complication related to the index procedure were reported, and no further arthroscopic procedures were necessary.

## Data Availability

The datasets generated during and/or analysed during the current study are available throughout the manuscript.

## Abbreviations

FAI: Femoroacetabular impingement
THA: Total hip arthroplasty
LCEA: Lateral centred edge angle
ROM: Range of motion
VAS: Visual analogic scale
NAHS: Non-arthritic hip score
iHOT-33: International Hip Outcome Tool
